# Russia's attacks on civilians strengthen Ukrainian resistance

**DOI:** 10.1093/pnasnexus/pgad386

**Published:** 2023-12-12

**Authors:** Henrikas Bartusevičius, Florian van Leeuwen, Honorata Mazepus, Lasse Laustsen, Andreas Forø Tollefsen

**Affiliations:** Department of Peace and Conflict Dynamics, Peace Research Institute Oslo, Hausmanns Gate 3, 0186 Oslo, Norway; Department of Social Psychology, Tilburg University, Professor Cobbenhagenlaan 225, 5037 DB Tilburg, The Netherlands; Department of Political Science, University of Amsterdam, Nieuwe Achtergracht 166, 1018 WV Amsterdam, The Netherlands; Department of Political Science and Centre for the Experimental-Philosophical Study of Discrimination, Aarhus University, Bartholins Allé 7, 8000 Aarhus C, Denmark; Department of Peace and Conflict Dynamics, Peace Research Institute Oslo, Hausmanns Gate 3, 0186 Oslo, Norway; Department of Sociology and Human Geography, University of Oslo, Moltke Moes vei 31, 0851 Oslo, Norway

**Keywords:** war, Ukraine, victimization, violence, resistance

## Abstract

The all-out Russian invasion of Ukraine commencing in February 2022 has been characterized by systematic violence against civilians. Presumably, the commanders of Russian forces believe that, for example, the bombing of residential buildings will force Ukrainians to lay down their arms. We ask whether military attacks against civilians deter or, in contrast, motivate resistance against the attackers. Two-wave probability surveys were collected in Ukraine in March and April 2022 (*N*s = 1,081 and 811, respectively). Preregistered analyses indicate that perceptions and experience of military attacks (victimization) did not decrease Ukrainians’ motivations to resist the invading forces. The analyses suggest that victimization positively relates to motivations to join military combat in defense positions. Military attacks against civilians are morally impermissible and prohibited under international humanitarian law. Our results suggest that such attacks are also counterproductive from a military perspective.

## Introduction

Since the start of the full-scale invasion of Ukraine in February 2022, the Russian forces have been systematically targeting civilians. Civilian victimization has taken various forms, from the bombing of apartment buildings to summary executions of ordinary citizens. The commanders of Russian forces presumably believed that imposing severe costs on the civilian population would force Ukrainians into surrender or at least extract strategic concessions. Examples of attempts to coerce governments by threatening their civilians abound in history (1). For example, during the Second World War, the bombing of Rotterdam and the threat of bombing Amsterdam contributed to the quick capitulation of the Netherlands. By contrast, although multiple cities were bombed in the United Kingdom as part of the Blitz, the British government did not surrender. Here, however, our focus is not on decisions of governments—we examine the experiences and motivations of the victimized civilians themselves.

Extant studies of insurgencies and rebellions show that violence against civilians produces heterogeneous effects, sometimes reducing resistance (e.g. 2) and sometimes increasing it (e.g. 3). Several studies suggest that violence against civilians gives rise to revenge motivations, which can lead to resistance when opportunities are present (4–7). Similarly, research on the 9/11 attacks has shown that victimization can evoke both confrontational responses (e.g. support for strong military response) and defensive responses aimed at self-protection (e.g. opposition to military action) (8).

Undoubtedly, extensive violence generates costs and fear among ordinary citizens. Yet, destroying people's property, injuring them, or killing their close ones can also result in intense revenge motivations. In theoretical accounts, revenge is often conceptualized as a strategy for deterring harms, occurring in response to actual harms (9). The strength of revenge motivations is upregulated by the levels of harm and subjective estimates that the aggressor will harm again in the future, and downregulated by subjective estimates of the future value of the relationship with the aggressor (ibid.). Given the immense costs of military attacks on Ukrainians and alienation from Russia, revenge motivations among victimized Ukrainians are thus likely to be strong. The effects of such emotions can be underestimated by observers or bystanders (10), and they may push people to engage in hazardous actions, even with high probability of injury and death.

Considering existing work, we make the following contributions. First, studies of insurgencies and rebellions have tended to analyze civilian victimization aggregated over some higher-level units (e.g. administrative regions); here, we focus on experiences and motivations reported by people themselves. Second, we analyzed data from an ongoing event, asking people to report their experiences at the time of the survey, rather than rely on memories of past events. Third, we focus on a major interstate war, exposing people both to extensive victimization and huge personal risks and costs associated with resistance. Finally, we utilized panel data collected over two waves, allowing us to conduct within-individual analyses that alleviate confounding concerns.

## Materials and methods

### Data

We conducted two waves of probability-based surveys in Ukraine during the first months of the invasion. Waves 1 and 2 were conducted, respectively, on March 9–12 and 2022 April 3–13. Both were administered online in Ukrainian and Russian by a local survey agency Info Sapiens. The agency aimed to generate a representative sample of the Ukrainian population aged 18–55 years in settlements with a minimum of 50,000 residents. However, some of the Ukrainians who fled beyond Ukraine's borders, or remained in areas of intense military combat, were likely not reached by the survey agency, which must be considered while evaluating our results. In wave 1, we surveyed 1,081 people. In wave 2, we aimed to recruit as many as possible from wave 1, stopping when the agency deemed collecting more responses was unrealistic. Roughly 75% (*N* = 811) of wave 1 respondents participated in wave 2. Following our preregistration, those who did not pass attention checks, as well as those who replied “prefer not to say” were excluded from analyses. This research was approved by the Ethics Review Board of the School of Social and Behavioral Sciences, Tilburg University (application: RP438). Informed consent was obtained from all respondents.

### Predictors

**Fig. 1.**
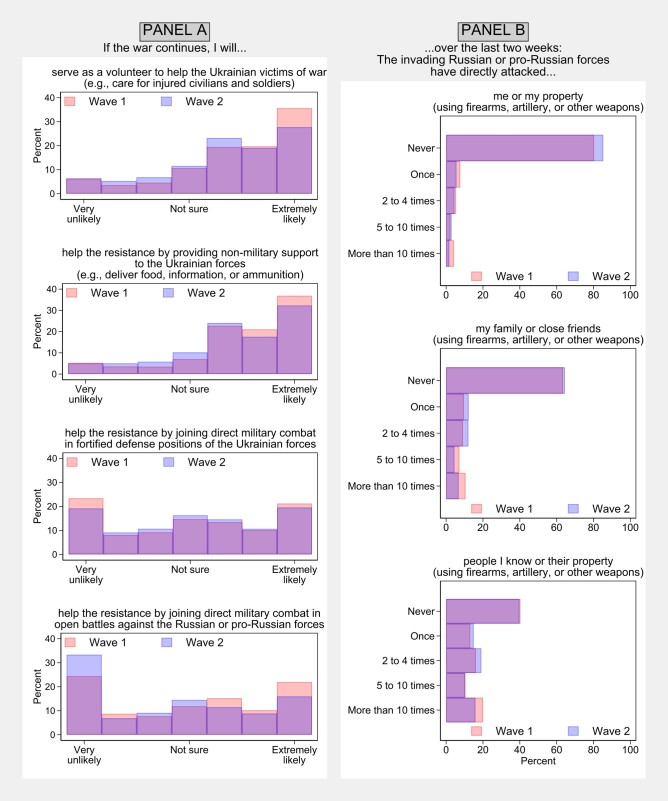
Descriptive statistics and formulations of questions used to measure outcome (Panel A) and predictor variables (Panel B). Both panels show percentage frequency distributions for all respondents of waves 1 and 2 who provided replies.

**Fig. 2.**
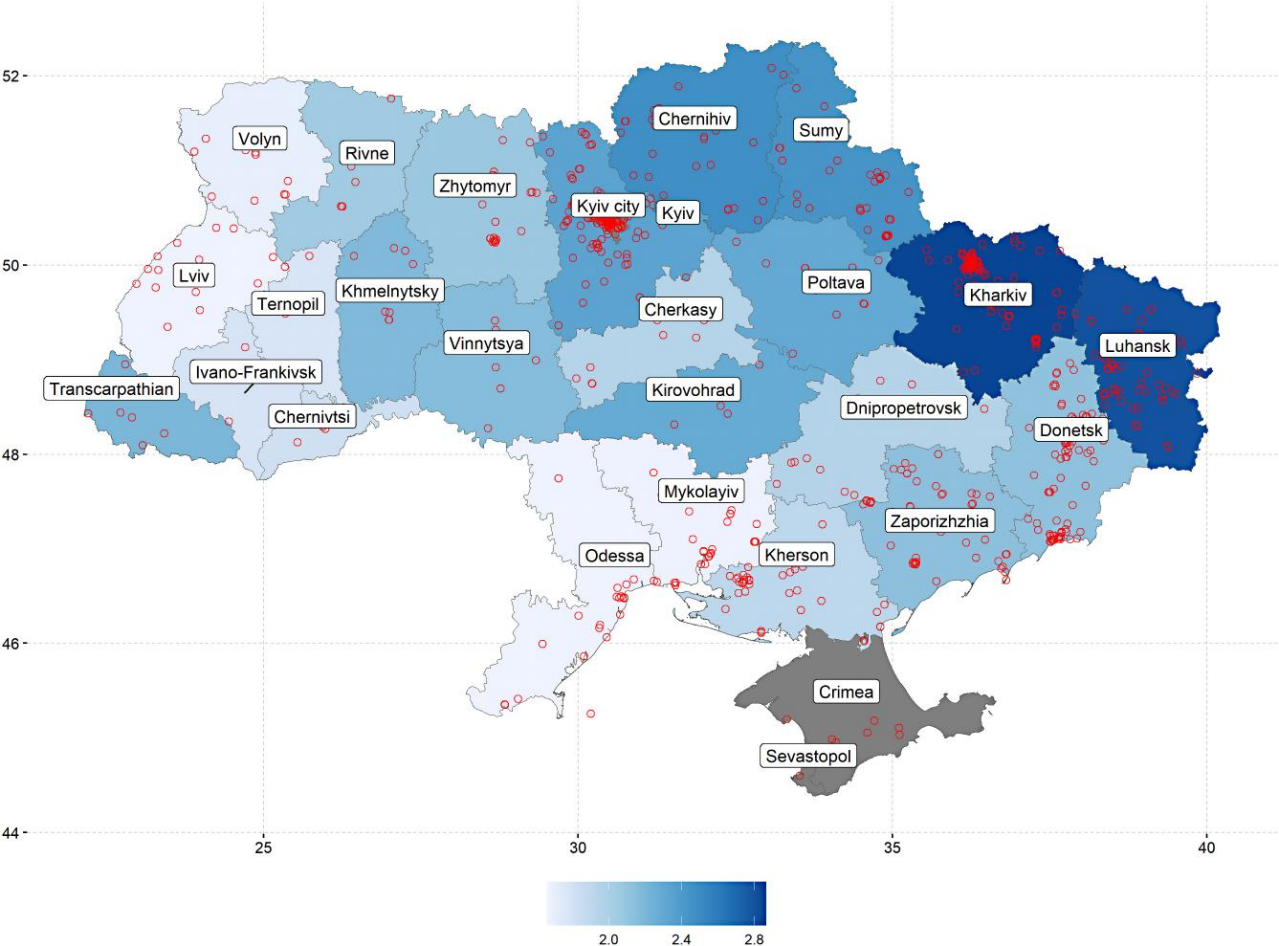
Self-reported frequency of attacks against family/friends overlayed by the incidence of attacks coded by the data project VIINA (11). Color coding represents self-reported scores (1 = never, 2 = once, 3 = 2 to 4 times, 4 = 5 to 10 times, 5 = more than 10 times) averaged over oblasts (administrative units). Circles represent VIINA-coded attacks (all types). Respondents in Crimea and Sevastopol were not accessed.

Respondents indicated on a five-point scale the frequency of attacks in the last 2 weeks against three targets: themselves, family and friends, and acquaintances (Figure 1 provides question formulations). As the main predictor, we used an average response to the three questions (Victimization scale VS) (Cronbach's *α*s = 0.72 and 0.67 in waves 1 and 2). We also analyzed the specific types of attacks as predictors (Victim self, victim family/friends, and victim acquaintances). As a validity check, we correlated the self-reported victimization data with the incidence of attacks in the respondents’ regions (oblasts) as provided by the data project Violent Incident Information from News Articles (VIINA), Version 1.0 (accessed Apr 21, 2023) (11). Figure 2 shows self-reported attacks against friends/family averaged over oblasts compared with the incidence of attacks (all types) in the oblasts from VIINA in the 2-week period before the start of wave 1, which correlated at *r* = 0.61.

### Outcomes

Respondents indicated on a seven-point scale the likelihood of future engagement in four types of resistance: volunteering to care for the victims of war; helping resistance logistics; joining military combat in defense positions; and joining military combat in open battles (Figure 1 provides question formulations). As the main outcome measure, we used an average response to the four questions (Resistance  Scale, RS). The scale showed satisfactory reliability: Cronbach's *α*s = 0.83 and 0.84 in waves 1 and 2. We also analyzed the specific types of resistance as outcomes. Although we also measured actual participation (finding corresponding results, which can be reproduced using Supplementary Material), our theoretical focus was on behavioral intentions, rather than reported behavior. Many Ukrainians fought for lack of other choices (e.g. they could not leave) or due to social pressure, and our theoretical focus was how victimization influences people's *motivations* to resist. However, research shows that what people say they intend to do correlates with what they do eventually (12). Studies suggest that intentions to engage in costly collective actions also correlate with actual behavior (13), and recent research reports a correlation between stated and actual sacrifices among frontline combatants (14). Furthermore, since we asked about intentions at the time of the survey and prospectively, our outcome measures alleviate reverse-causality concerns.

### Modeling

We preregistered two types of analyses: multiple regressions, which used between-individual variation at wave 2 to estimate the coefficients (hereafter, between-individual models); and two-way linear fixed-effects regressions with predictors and outcomes measured at both waves, which utilized within-individual variation over time, from waves 1 to 2 (within-individual models). Between-individual models are vulnerable to confounding by individual differences, such as gender; thus, we controlled for a basic set of controls: age, gender, and education. Within-individual models estimate whether within-individual changes in the predictors relate to within-individual changes in the outcomes. Because most individual traits remain constant over short periods of time, their influence on the within-individual variation is controlled for. Hence, these models fully account for observed and unobserved time-invariant characteristics. As such these models do not require controlling for stable individual-level traits. To aid the interpretation of coefficients, we rescaled all variables to range from 0 to 1.

## Results

At the start of the invasion, we reported descriptive statistics from wave 1 in a blog post (15), revealing extensive victimization of Ukrainians and their strong motivations to resist the invading forces (see also Figure 1). The data also preliminarily suggested a link between victimization and resistance intentions. The present analyses corroborate this link. Specifically, between-individual analyses of wave 1 data revealed that VS positively predicted RS, *b*  *=* 0.2, 95% CI = [0.14, 0.25], *P* < 0.001. Furthermore, each subtype of victimization positively predicted each subtype of resistance (*P*s < 0.01). Wave 1 analyses were not preregistered. Analogous preregistered analyses of wave 2 data revealed similar results, with VS positively predicting RS, *b* = 0.19, 95% CI = [0.12, 0.27], *P* < 0.001, and—except for Victim self—the subtypes of victimization predicting all types of resistance intentions. The more conservative (preregistered) analyses, which exclusively used within-participant variation to estimate the coefficients, also revealed a positive but weaker association between VS and RS, *b* = 0.05, 95% CI = [0.00, 0.11], *P* = 0.062. Disaggregated analyses indicated that this relationship was driven by one outcome: intentions to join military combat in defense positions, *b* = 0.09, 95% CI = [0.01, 0.16], *P* = 0.027. We also explored potential suppressors of these effects (see preregistration). Theoretical accounts of revenge suggest that revenge motivations subside after the revenge is taken (9). Hence, those Ukrainians who experienced military attacks at wave 1, and then joined the resistance, may had reduced intentions to engage in further resistance at wave 2. The panel analyses revealed that including self-reported resistance as a covariate produced similar associations of VS with both RS, *b* = 0.06, 95% CI = [0.00, 0.11], *P* = 0.051, and intentions to resist in defense positions, *b* = 0.09, 95% CI = [0.02, 0.17], *P* = 0.017. Detailed regression results can be reproduced using the code and data in Supplementary Material.

## Discussion

Attacks on civilians by the Russian forces are puzzling, given the shortage of ammunitions and Russia's difficulties in advancing into or upholding Ukrainian territories by directly engaging Ukraine's military forces. One explanation for why scarce military resources have been used against benign nonmilitary targets is the Kremlin's belief that terrorizing civilians will extract political concessions. A recent study (16) suggests, however, that Ukrainians reject political or territorial concessions regardless of costs, including civilian fatalities. Another explanation for the victimization of Ukrainian civilians is the belief that doing so will deter their resistance. The present research shows, however, that military attacks did not decrease Ukrainians’ motivations to fight. In contrast, resistance motivations appear to increase as a function of victimization, especially when it comes to combat in defense positions. The stronger association with intentions to resist in defense positions appears in line with extant work on revenge motivations: although the psychology regulating revenge exerts a powerful force on behavior, it likely remains sensitive to potential costs of, and risks associated with, enacting revenge.

Taken together, by targeting civilians, the Russian forces not only violate international humanitarian law and expose themselves to international condemnation, but also likely generate new recruits or defenders for the adversary they are fighting.

## Supplementary Material

pgad386_Supplementary_Data

## Data Availability

The data, code, and questionnaires required to reproduce the results are available in Supplementary Material.
