# Supplementary material for: Russia's attacks on civilians strengthen Ukrainian resistance
Source: PNAS Nexus. 2023 Dec 12;2(12):pgad386. doi: 10.1093/pnasnexus/pgad386 (PMC10715192; doi:10.1093/pnasnexus/pgad386)
Supplement: pgad386_Supplementary_Data [file pgad386_supplementary_data.zip › w2_questionnaire_final (pre_trans)_SM.pdf]

Study: Citizens’ responses to war in Ukraine

Wave 2

Q	**Survey element & Instruction**	English version [the survey will be translated to Ukrainian and Russian; none of the questionnaires will be administered in English]
	Language	Please choose language:<br>1) Ukrainian<br>2) Russian
1	Introduction	Dear Participant,<br><br>As the war in Ukraine continues and your situation may have changed over the last weeks, we are re-contacting you to ask about your views and experiences. We do not have access to any of your identifying information. The survey company [anonymized for the review process] is in charge of forwarding the questionnaire to you.<br><br>We appreciate your time and willingness to participate!<br><br>This study is conducted by [anonymized for the review process].<br><br>**Aims of the Study:**<br>The purpose of this study is to gain knowledge of how people experience conflict and how this influences how they feel and what they want.<br><br>**Eligibility Requirements:**<br>You are welcome to take part in the study if you are over 18.<br><br>**What you will need to do and time commitment:**<br>We will ask you questions about what you experienced since the start of the Russian invasion on 24 February 2022, your views on peace negotiations, and what you would do if the war continues. The study will take around 10 minutes.<br><br>**Voluntariness of participation**<br>We ask your consent to participate in this study. Participating in this research is voluntary. You have the right not to take part in this study. If you decide to participate in this study, you are free to withdraw from this study at any time, without any negative consequences, and without giving any reason. You are free to only answer questions that you want to reply to.<br><br>**Risks involved in participating:**<br>Risks are minimal for participating in this study. However, we understand that you might be currently under distress and experiencing a difficult situation. As we ask you to reflect on the war and emotions you’ve been experiencing, some questions might make you feel uncomfortable. Please remember that you can quit your participation at any moment.<br><br>**Your data:**<br>The anonymized research data will be stored safely for a period of 10 years. Only the researchers have access to this data. When the results

		of this study are published or presented at conferences, no information will be presented that can reveal your personal identity. Anonymized data collected in this study might be useful for future research and therefore this data will be anonymously available.<br><br>**Reward/Reimbursement**<br>For participating in this study you will be rewarded according to your agreement with the survey agency that has invited you to do this survey.<br><br>**Ethical Approval**<br>This study was approved by the ethics review board [anonymized for the review process].<br><br>**Participation**<br>If you decide to participate in this study, you can click on 'accept' and continue to the study.<br><br>**Contact**<br>Any questions about this study can be directed to:<br>[anonymized for the review process]
2	Consent	Please read and confirm the following statements:<br>• I confirm that I have read and understand the information sheet for the above study.<br>• I understand that my participation is voluntary and that I am free to withdraw at any time without giving any reason.<br>• I understand that my data will be treated confidentially and any publication resulting from this work will report only data that does not identify me. My anonymised responses, however, may be shared with other researchers or made available in online data repositories.<br>• I freely agree to participate in this study.<br><br>Choice:<br>Do not accept –> Thank you screen<br>Accept → Begin the study
3	Age<br>Please measure the exact age	How old are you?
4	Region<br><br>[administrative units: pre-set oblasts]	What region do you currently stay in?<br><br>Please provide 'prefer not to say option'<br><br>Provide: 'outside of Ukraine' as an option?
5	Region<br><br>[administrative units: pre-set oblasts]	What region did you live in before the Russian invasion on 24 February 2022?<br><br>Please provide 'prefer not to say option'

6	Region	Is the region that you are in now, the same as the one you were in two weeks ago?<br><br>Yes; No; Prefer not to say
7	Intensity/severity of the war	Below are some questions about your experiences over the last two weeks.<br><br>To what extent is the municipality you are currently in under the attack of the Russian and pro-Russian forces? Please indicate your answer from 1- 'Has not been attacked at all' to 7- 'Has been heavily attacked'
	Two blocks will follow below Block 1 and Block 2<br><br>Please program the survey, so that the order of block 1 and 2 is randomized. Half of the respondents first do block 1 and then block 2 and half of the respondents first do block 2 and then block 1. Make sure that the data file includes a variable that shows for each respondent which block they did first.	
8	Block 1: Conflict perception	Please tell us how often the events described below have happened over the last two weeks:<br><br>1. The invading Russian or pro-Russian forces have directly attacked me or my property (using firearms, artillery, or other weapons)<br>2. The invading Russian or pro-Russian forces have directly attacked my family or close friends, or their property (using firearms, artillery, or other weapons)<br>3. The invading Russian or pro-Russian forces have directly attacked people I know or their property (using firearms, artillery, or other weapons)<br><br>For each statement:<br>*If your locality was damaged by bombs or attacks, but you were not personally attacked or your property was not damaged, please answer "never"<br><br>Answer options:<br>0 = Never<br>1 = Once<br>2 = 2 to 4 times<br>3 = 5 to 10 times<br>4 = More than 10 times<br>998 = Prefer not to say
9	Block 2: Conflict perception	For each of the following statements, please indicate the likelihood that you will engage in the described activity:<br><br>1. If the war continues, I will serve as a volunteer to help the Ukrainian victims of war (e.g., care for injured civilians and soldiers)<br>2. If the war continues, I will help the resistance by providing non-military support to the Ukrainian forces (e.g., deliver food, information, or ammunition)<br>3. If the war continues, I will help the resistance by joining direct military combat in fortified defense positions of the Ukrainian forces<br>4. If the war continues, I will help the resistance by joining direct military combat in open battles against the Russian or pro-Russian forces<br><br>Answer options:

		0 = Very unlikely<br>1 = Moderately unlikely<br>2 = Somewhat unlikely<br>3 = Not sure<br>4 = Somewhat likely<br>5 = Moderately likely<br>6 = Extremely likely<br>988 = Prefer not to say
10	Engagement in resistance	For each of the following statements, please indicate if in the last two weeks you have engaged in the described activity:<br>1. I served as a volunteer to help the Ukrainian victims of war (e.g., cared for injured civilians and soldiers)<br>2. I helped the resistance by providing non-military support to the Ukrainian forces (e.g., delivered food, information, or ammunition)<br>3. I helped the resistance by joining direct military combat in fortified defense positions of the Ukrainian forces<br>4. I helped the resistance by joining direct military combat in open battles against the Russian or pro-Russian forces<br><br>0 = No; 1 = Yes, once; 2 = Yes, several times; 3 = Yes, many times; 998 = Prefer not to say
11	Emotions frequency<br><br>Please present the statements in random order.	Please tell us how often in the past week you were feeling...<br><br>1. Afraid<br>2. Frightened<br>3. Scared<br>4. Angry<br>5. Hostile<br>6. Disgusted<br>7. Sad<br>8. Lonely<br>9. Downhearted<br>10. Proud<br>11. Strong<br>12. Confident<br><br>Answer options:<br>0 = Never<br>1 = Very rarely<br>2 = Rarely<br>3 = Sometimes<br>4 = Often<br>5 = Very often<br>6 = All the time<br>998 = Prefer not to say

12	Leadership preference measure<br><br>Please present the statements in random order	C1: Imagine that you need to elect a new leader of your country right now. What kind of leader would you prefer to lead your country?<br><br>1. I would like a leader who is competent<br>2. I would like a leader who is trustworthy<br>3. I would like a leader who is dominant<br>4. I would like a leader who is generous<br>5. I would like a leader who is strong<br>6. I would like a leader who is warm<br>7. I would like a leader who is tough-minded<br><br>Scale:<br><br>- Strongly Disagree (1)<br>- Disagree (2)<br>- Somewhat Disagree (3)<br>- Neither Agree nor Disagree (4)<br>- Somewhat Agree (5)<br>- Agree (6)<br>- Strongly Agree (7)<br>998 = Prefer not to say
13	Identity 1<br><br>Please present the questions in random order	Below is a set of questions related to your personal identification.<br><br>Please answer each of the questions on the scale from 1- Not at all to 7- Very strongly<br><br>1. How strongly do you identify with Ukrainians?<br>2. How strongly do you identify with Russians?<br>3. How strongly do you identify with Europeans?<br><br>Scale:<br><br>1.Not at all<br>2.<br>3.<br>4.<br>5.<br>6.<br>7. Very strongly<br>998 = Prefer not to say
14	Identity 2	Please answer each of the questions on the scale from 1- Not close at all 7- Very close

	Please present the questions in random order	1. How close do you feel to Ukrainians?<br>2. How close do you feel to Russians?<br>3. How close do you feel to Europeans?<br><br>Scale:<br><br>1. Not close at all<br>2.<br>3.<br>4.<br>5.<br>6.<br>7. Very close<br>998 = Prefer not to say
15	Socioeconomic status	Think of a ladder with 10 steps representing where people stand in Ukraine. At step 10 are people who are the best off—those who have the most money, the most education, and the most respected jobs. At step 1 are the people who are worst off—those who have the least money, least education, and the least respected jobs or no job. Where would you place yourself on this ladder?<br><br>1. Bottom of the ladder<br>2<br>3<br>4<br>5<br>6<br>7<br>8<br>9<br>10. Top of the ladder<br>998 = Prefer not to say
16	Good neighbor	Please indicate your agreement with the following statement: 10 years from now, Russia can become a good neighbour to Ukraine.<br><br>Reply options:<br>0 = Strongly disagree; 6 = Strongly agree
17	Peace agreement (1)	In order for the current peace talks to be successful, some people think the Ukrainian government should make concessions to Russia. To what extent do you agree with the following:<br><br>1. Ukraine should cede sovereignty over Crimea<br>2. Ukraine should cede sovereignty over the territories of Donbas (DPR/LPR) occupied before 2022<br>3. Ukraine should cede sovereignty over the entire territory of Donbas

		4. Ukraine should accept to be neutral if acceptable security guarantees can be provided by third countries.<br>5. Ukraine should refrain from becoming member of the EU.<br><br>Reply options:<br>0 = Strongly disagree; 6 = Strongly agree; -99 = Don't know
18	Peace agreement (2)	To what extent do you agree with the following statements:<br><br>1. There should be an immediate cease-fire at this point<br>2. A peace agreement should be put to a referendum in Ukraine<br>3. The Ukraine Delegation to peace talks should include women representatives<br><br>Reply options:<br>0 = Strongly disagree; 6 = Strongly agree; -99 = Don't know
19	Food insecurity	In the past 7 days, on how many days…<br><br>1. You did not have enough food?<br>2. You had to limit the portion size of meals?<br>2. You did not have enough potable liquids (water, tea, etc.) to drink?<br><br>Reply options:<br>0, 1, 2, 3, 4, 5, 6, 7
20	Information (a)	What is **the main source** of information you rely on to be updated about the ongoing war?<br><br>1. TV or radio, Ukrainian channels<br>2. TV or radio, Russian channels<br>3. TV or radio, European or other channels<br>4. Press (print or online), Ukrainian outlets<br>5. Press (print or online), Russian outlets<br>6. Press (print or online), European or other outlets<br>7. Social media<br>8. Other, please fill in:<br>9. Prefer not to say<br><br>[One choice possible only]
21	Information (b)	I trust that the information I have access to about the war is accurate:<br><br>Reply options:<br>0 = Strongly disagree; 6 = Strongly agree

	Two blocks will follow below Block 1 and Block 2<br>Please program the survey, so that the order of block 1 and 2 is randomized. Half of the respondents first do block 1 and then block 2 and half of the respondents first do block 2 and then block 1. Make sure that the data file includes a variable that shows for each respondent which block they did first.	
22	Block 1<br><br>Sexual violence	Please tell us how many of the events described below you have heard about since the start of the Russian invasion on 24 February 2024:<br>1. The invading Russian or pro-Russian forces have committed acts of rape or sexual assault against civilians<br>2. The invading Russian or pro-Russian forces have committed acts of rape or sexual assault against people you know<br><br>Reply options:<br>0 = None; 1 = One; 2 = 2 to 4; 3 = 5 to 10; 4 = More than 10; 998 = Prefer not to say
23	Block 2<br><br>Migration intentions questions	1. How likely is it that you will leave during the next month?<br>Reply options:<br>0 = very unlikely, 6 = extremely likely<br><br>2. If leaving this location, where would you go?<br>Reply options:<br>0 = Neighboring Oblast, 1 = Other area in Ukraine, 2 = Russia, 3 = Belarus, 4 = Moldova, 5 = Romania, 6 = Hungary, 7 = Slovakia, 8 = Poland, 9 = Country not listed
24		This is an attention check. Please pick 'Green' from the list of colors below:<br><br>1) Red<br>2) Blue<br>3) Green<br>4) Orange<br>5) Brown
25	Comments<br>[open question]	This is the last question.<br>Do you have any comments that you would like to share with us?
26	Debriefing	Thank you very much for your participation.<br><br>The purpose of this research project is to examine what people experience during a military attack, how they respond, what are their preferences for leaders, and what they think about the peace negotiations.<br><br>Thank you for your participation in our study. If you have any questions about the survey please contact [anonymized for the review process]<br><br>[anonymized for the review process] will also disseminate the findings of this study using Twitter account [anonymized for the review process] and through the channels of [anonymized for the review process].<br><br>In case there are any remaining questions, please feel free to contact me.<br><br>Again thank you very much for your time, participation and effort.<br>[anonymized for the review process]
