# Supplementary material for: Russia's attacks on civilians strengthen Ukrainian resistance
Source: PNAS Nexus. 2023 Dec 12;2(12):pgad386. doi: 10.1093/pnasnexus/pgad386 (PMC10715192; doi:10.1093/pnasnexus/pgad386)
Supplement: pgad386_Supplementary_Data [file pgad386_supplementary_data.zip › w1_questionnaire_final (pre_trans)_SM.pdf]

Study: Citizens' responses to war in Ukraine

Dr. Honorata Mazepus (Leiden Univesrity)*, Dr. Florian van Leeuwen (Tilburg University), Prof. Mark van Vugt (Vrije Universiteit Amsterdam), Dr. Lasse Lausten (Aarhus University), Dr. Alexander Bor (Aarhus University), and Dr. Henrikas Bartusevičius (Peace Research Institute Oslo)

*contact: h.mazepus@fgga.leidenuniv.nl

Q	Survey element & Instruction	English	Ukrainian	Russian
1	Introduction	Dear Participant,<br><br>Welcome to this study. We appreciate your time and willingness to share your views and experiences.<br><br>This study is conducted by Dr. Honorata Mazepus (h.mazepus@fgga.leidenuniv.nl) from Leiden University (Netherlands) in collaboration with researchers from Tilburg University, Vrije Universiteit Amsterdam (Netherlands), Aarhus University (Denmark) and Peace Research Institute Oslo (Norway).<br><br>**Aims of the Study:**<br>The purpose of this study is to gain knowledge of how people experience conflict and how this influences how they feel and what they want.<br><br>**Eligibility Requirements:**<br>You are welcome to take part in the study if you are over 18.<br><br>**What you will need to do and time commitment:**<br>We will ask you questions about what you experienced since the start of the Russian invasion, what your preferences are for the political system of your country, and what you would do if the war continues. The study will take around 15 minutes.	**Цілі дослідження:**<br><br>**Вимоги до участі:**<br>Ви можете взяти участь у дослідженні, якщо вам більше 18 років.<br><br>**Що вам потрібно буде зробити і скільки часу для цього знадобиться:**<br>Дослідження займе близько 15 хвилин.<br><br>**Ризики, пов'язані з участю:**<br><br>**Конфіденційність ваших даних:**	**Цели исследования:**<br><br>**Условия участия:**<br>Вы можете принять участие в исследовании, если вам больше 18 лет.<br><br>**Что вам нужно будет сделать и сколько времени для этого понадобится:**<br>Исследование займет около 15 минут.<br><br>**Риски, связанные с участием:**

		**Voluntariness of participation**<br>We ask your consent to participate in this study. Participating in this research is voluntary. You have the right not to take part in this study. If you decide to participate in this study, you are free to withdraw from this study at any time, without any negative consequences, and without giving any reason. You are free to only answer questions that you want to reply to.<br><br>**Risks involved in participating:**<br>Risks are minimal for participating in this study. However, we understand that you might be currently under distress and experiencing a difficult situation. As we ask you to reflect on the war and emotions you've been experiencing, some questions might make you feel uncomfortable. Please remember that you can quit your participation at any moment.<br><br>**Your data:**<br>The anonymized research data will be stored safely for a period of 10 years. Only the researchers have access to this data. When the results of this study are published or presented at conferences, no information will be presented that can reveal your personal identity. Anonymized data collected in this study might be useful for future research and therefore this data will be anonymously available.<br><br>**Reward/Reimbursement**<br>For participating in this study you will be rewarded according to your agreement with the survey agency that has invited you to do this survey.<br><br>**Ethical Approval**		

		This study was approved by the ethics review board (ERB) of Tilburg University in the Netherlands under number [enter ERB number here].<br><br>**Participation**<br>If you decide to participate in this study, you can click on 'accept' and continue to the study.<br><br>**Contact**<br>Any questions about this study can be directed to:<br>Dr. Honorata Mazepus<br>h.mazepus@fgga.leidenuniv.nl		
2	Consent	Please read and confirm the following statements:<br>• I confirm that I have read and understand the information sheet for the above study.<br>• I understand that my participation is voluntary and that I am free to withdraw at any time without giving any reason.<br>• I understand that my data will be treated confidentially and any publication resulting from this work will report only data that does not identify me. My anonymised responses, however, may be shared with other researchers or made available in online data repositories.<br>• I freely agree to participate in this study.<br><br>Choice:<br>Do not accept –> Thank you screen<br>Accept → Begin the study	Будь ласка, прочитайте та підтвердьте наступні положення:<br>• Я підтверджую, що прочитав та розумію інформаційний лист щодо вищевказаного дослідження.<br>• Я розумію, що моя участь є добровільною і що я можу відмовитись від участі у будь-який час, не вказавши жодної причини.<br>• Я розумію, що мої дані будуть оброблятися конфіденційно, і будь-яка публікація за результатами цього дослідження міститиме лише дані, за якими мене неможливо ідентифікувати. Однак мої анонімні відповіді можуть бути передані іншим дослідникам або доступні в Інтернет-сховищах даних.<br>• Я добровільно погоджуюсь брати участь у цьому дослідженні.	Пожалуйста, прочитайте и подтвердите следующие положения:<br>• Я подтверждаю, что прочитал и понимаю информационное письмо по вышеуказанному исследованию.<br>• Я понимаю, что мое участие является добровольным и что я могу отказаться от участия в любое время, не указав никаких причин.<br>• Я понимаю, что мои данные будут обрабатываться конфиденциально, и любая публикация по результате этого исследования будет содержать только данные, по которым меня невозможно идентифицировать. Однако мои анонимные ответы могут быть переданы другим исследователям или доступны в Интернет-хранилищах данных.<br>• Я даю добровольное согласие участвовать в этом исследовании
3	Gender	What is your gender:<br>1- Male<br>2- Female<br>3- Prefer not to say	Відмітьте вашу стать<br>1 – чоловіча<br>2 – жіноча<br>3 – волію не відповідати на це питання	Вы:<br>1- Мужчина<br>2- Женщина<br>3 - предпочитаю не отвечать на этот вопрос
4	Age	How old are you?	Відмітьте ваш вік	Сколько вам лет?

5	Region<br><br>[administrative units]	What region do you live in/lived in before the invasion?<br><br>Please provide ‘prefer not to say option’		
6	Education level<br><br>[please check if these categories make sense in Ukraine]	What is the highest level of education that you have completed?<br>- Primary<br>- High school graduate<br>- Professional-technical (vocational)<br>- Incomplete higher<br>- Bachelor degree<br>- Master degree<br>- Doctorate<br>- 988 = Prefer not to say	Який найвищий рівень освіти Ви здобули?<br>- базова загальна<br>- повна загальна<br>- професійно-технічна<br>- неповна вища<br>- базова вища (бакалавр)<br>- повна вища (магістр)<br>- науковий ступінь<br>- 988 = волію не відповідати на це питання	Какой самый высокий уровень образования вы получили?<br>- базовое общее<br>- полное общее<br>- профессионально-техническое<br>- неполное высшее<br>- базовое высшее (бакалавр)<br>- полное высшее (магистр)<br>- научная степень<br>- 988 = предпочитаю не отвечать на этот вопрос
7	Intensity/severity of the war	Below are some questions about your experiences since the start of the Russian invasion on 24 February 2022.<br><br>To what extent is the municipality you are currently in under the attack of the Russian and pro-Russian forces? Please indicate your answer from 1- ‘Has not been attacked at all’ to 7- ‘Has been heavily attacked’		
8	Armed Forces	Have you already joined Ukrainian Armed Forces or local territorial defence units?<br><br>1- Yes<br>2- No<br>3- Prefer not to say		
	Two blocks will follow below Block 1 and Block 2<br><br>Please program the survey, so that the order of block 1 and 2 is randomized. Half of the respondents first do block 1 and then block 2 and half of the respondents first do block 2 and then block 1. Make sure that the data file includes a variable that shows for each respondent which block they did first.			
9	Block 1: Conflict perception	Please tell us how often the events described below have happened since the start of the Russian invasion.<br><br>1.The invading Russian or pro-Russian forces have directly attacked me or my property (using firearms, artillery, or other weapons).		

		2.The invading Russian or pro-Russian forces have directly attacked my family or close friends, or their property (using firearms, artillery, or other weapons).<br>3.The invading Russian or pro-Russian forces have directly attacked people I know or their property (using firearms, artillery, or other weapons).<br><br>Answer options:<br>0 = Never<br>1 = Once<br>2 = 2 to 4 times<br>3 = 5 to 10 times<br>4 = More than 10 times<br>998 = Prefer not to say		
10	Block 2: Conflict perception	For each of the following statements, please indicate the likelihood that you will engage in the described activity:<br>1. If the war continues, I will serve as a volunteer to help the Ukrainian victims of war (e.g., care for injured civilians and soldiers).<br>2. If the war continues, I will help the resistance by providing non-military support to the Ukrainian forces (e.g., deliver food, information, or ammunition).<br>3. If the war continues, I will help the resistance by joining direct military combat in fortified defense positions of the Ukrainian forces.<br>4. If the war continues, I will help the resistance by joining direct military combat in open battles against the Russian or pro-Russian forces.<br><br>Answer options:<br>0 = Very unlikely<br>1 = Moderately unlikely<br>2 = Somewhat unlikely<br>3 = Not sure<br>4 = Somewhat likely<br>5 = Moderately likely		

		6 = Extremely likely<br>988 = Prefer not to say		
11	Emotions frequency<br><br>Please present the statements in random order.	Please tell us how often in the past week you were feeling...<br><br>1. Afraid<br>2. Frightened<br>3. Scared<br>4. Angry<br>5. Hostile<br>6. Disgusted<br>7. Sad<br>8. Lonely<br>9. Downhearted<br>10. Proud<br>11. Strong<br>12. Confident<br><br>Answer options:<br>0 = Never<br>1 = Very rarely<br>2 = Rarely<br>3 = Sometimes<br>4 = Often<br>5 = Very often<br>6 = All the time<br>998 = Prefer not to say		
12	Experiment intro text	On the next page we ask you to carefully read a short text. It is very important that you read the text, since the following questions relate directly to it. Click next to read the text.		
13	Experiment<br><br>Please assign participants randomly to one of the two conditions so they either see C1 or C2. Please make sure that for each	C1:<br>Imagine that you need to elect a new leader of your country right now. What kind of leader would you prefer to lead your country?<br><br>C2:<br>Imagine that peace and quiet returns to your country and that the neighbors no longer		

	participant it is recorded who read C1 and who read C2	threaten Ukraine. What kind of leader would you prefer to lead your country?		
14	Leadership preference measure<br><br>Please present on the same page as the C1 or C2 text<br><br>Please present the statements in random order	1. I would like a leader who is competent<br>2. I would like a leader who is trustworthy<br>3. I would like a leader who is dominant<br>4. I would like a leader who is generous<br>5. I would like a leader who is strong<br>6. I would like a leader who is warm<br>7. I would like a leader who is tough-minded<br><br>Scale<br><br>- Strongly Disagree (1)<br>- Disagree (2)<br>- Somewhat Disagree (3)<br>- Neither Agree nor Disagree (4)<br>- Somewhat Agree (5)<br>- Agree (6)<br>- Strongly Agree (7)<br>998 = Prefer not to say	1. Я хотів/ла би мати компетентного лідера<br>2. Я хотів/ла би мати лідера, якому можна довіряти<br>3. Я хотів/ла би мати лідера, який домінує<br>4. Я хотів/ла би мати щедрого лідера<br>5. Я хотів/ла би мати сильного лідера<br>6. Я хотів/ла би мати приємного лідера<br>7. Мені б хотілося, щоб лідер був жорстким<br><br>- Категорично не згоден/на (1)<br>- Не згоден/на (2)<br>- Дещо не згоден/на (3)<br>- Ані згоден/на, ані не згоден/на (4)<br>- Дещо згоден/на (5)<br>- Погоджуюсь (6)<br>- Цілком погоджуюсь (7)<br>988 = волію не відповідати на це питання	1. Я хотел/-ла бы компетентного лидера<br>2. Я хотел/-ла бы лидера, которому можно доверять<br>3. Я хотел/-ла бы лидера, который доминирует<br>4. Я хотел/-ла бы щедрого лидера<br>5. Я хотел/-ла бы сильного лидера<br>6. Я хотел/-ла бы теплого, душевного лидера<br>7. Я хотел/-ла бы жесткого лидера<br><br>- Совершенно не согласен/-на (1)<br>- Не согласен/-на (2)<br>- В некоторой степени не согласен/-на (3)<br>- Ни согласен/-на, ни не согласен/-на (4)<br>- В некоторой степени согласен/-на (5)<br>- Согласен/-на (6)<br>- Полностью согласен/-на (7)<br>988 = предпочитаю не отвечать на этот вопрос
15	Current leader<br><br>Please present the statements in random order	We now ask you to think about the **current leader** of Ukraine. Please indicate for each statement below, the extent to which it accurately describes the current leader of your country using the presented scale. On this scale '1' reflects that a statement 'not at all'<br>describes your current leader. Likewise, '4' indicates that a statement describes your current leader 'somewhat well', while '7' reflects that the statement describes your current leader 'very much':<br><br>1. Zalensky is competent<br>2. Zalensky is trustworthy	Тепер ми просимо Вас подумати про **поточного лідера** України. Будь ласка, вкажіть для кожної заяви нижче, наскільки вона точно описує поточного лідера Вашої країни, використовуючи представлену шкалу. На цій шкалі «1» відображає, що твердження «зовсім не» описує Вашого нинішнього очільника країни. Так само «4» вказує на те, що висловлювання описує вашого поточного лідера «певною мірою», тоді як «7» відображає, що твердження, яке описує вашого поточного лідера «цілком відповідає дійсності»<br><br>1. Заленський – компетентний лідер	Теперь мы просим Вас подумать о **нынешнем лидере** Украины . Используя представленную ниже шкалу, для каждого утверждения укажите, насколько точно оно описывает нынешнего лидера Вашей страны. По этой шкале "1" отражает, что утверждение "совсем не описывает" Вашего нынешнего лидера. Аналогичным образом, "4" означает, что утверждение описывает вашего нынешнего лидера "в некоторой степени", а "7" означает, что утверждение описывает Вашего нынешнего лидера "очень хорошо".<br><br>1. Заленский – компетентный лидер

		3. Zalensky is dominant<br>4. Zalensky is generous<br>5. Zalensky is strong<br>6. Zalensky is warm<br>7. Zalensky is tough-minded<br><br>Scale<br>Not at all (1)<br>(2)<br>(3)<br>Somewhat (4)<br>(5)<br>(6)<br>- Very much (7)<br>988 = Prefer not to say	2. Заленський лідер, який заслуговує на довіру<br>3. Заленський – домінуючий лідер<br>4. Заленський – щедрий лідер<br>5. Заленський – сильний лідер<br>6. Заленський – приємний теплий лідер<br>7. Заленський — жорсткий лідер<br><br>Зовсім не так (1)<br>(2)<br>(3)<br>Певною мірою так (4)<br>(5)<br>(6)<br>Цілком відповідає дійсності (7)<br>988 = волію не відповідати на це питання	2. Заленский лидер, заслуживающий доверия<br>3. Заленский – доминирующий лидер<br>4. Заленский – щедрый лидер<br>5. Заленский – сильный лидер<br>6. Заленский – душевной теплый лидер<br>7. Заленский – жесткий лидер<br><br>Совсем нет (1)<br>(2)<br>(3)<br>В некоторой степени (4)<br>(5)<br>(6)<br>- Очень (7)<br>988 = предпочитаю не отвечать на этот вопрос
16	Strong leader preference	Please indicate to what extent do you agree with the following statements, from 1- strongly disagree to 7- strongly agree.<br><br>1. Our country needs a strong leader right now<br>2. We need strong leadership in order to make -this society survive<br>3. We need strong leadership in order to overcome societies' difficulties<br><br>- Strongly Disagree (1)<br>- Disagree (2)<br>- Somewhat Disagree (3)<br>- Neither Agree nor Disagree (4)<br>- Somewhat Agree (5)<br>- Agree (6)<br>- Strongly Agree (7)<br>998 = Prefer not to say		
17	Trust in institutions<br><br>Please present the statements in random order	Please indicate to what extent you trust the following institutions. Please use the scale from 1- Not at all to 7- Fully<br><br>1. President of Ukraine<br>2. Parliament of Ukraine	Будь ласка, вкажіть, наскільки Ви довіряєте наступним установам в Україні. Будь ласка, використовуйте шкалу від 1- «Зовсім не довіряю» до 7- «Повністю довіряю»<br><br>1- Президент	Пожалуйста, укажите, насколько Вы доверяете следующим учреждениям в Украине. Пожалуйста, используйте шкалу от 1 – «совсем не доверяю» до 7 – «полностью доверяю»

		3. National government of Ukraine<br>4. Local government in your region<br>5. Courts in Ukraine<br>6. The European Union	2- Верховна Рада<br>3- Кабінет Міністрів<br>4- Місцева влада<br>5- Суд<br>6- Європейський Союз	1. Президент<br>2. Парламент<br>3. Центральные власти<br>4. Местные власти<br>5. Суд<br>6. Европейский Союз
18	Political system preferences<br><br>Please present the statements in random order	Please indicate to what extent you agree or disagree with the following statements from 1- 'Fully disagree' to 7- 'Fully agree'<br><br>1. It is important that courts treat everyone the same<br>2. Citizens should have the right to pick their political representatives in free and fair elections<br>3. It is NOT important that opposition parties are free to criticise the government<br>4. Judges should be independent from elected politicians<br>5. It is important that the media are free to criticise the government<br>6. It is NOT important that the courts are able to stop the government acting beyond its authority<br>7. Democracy has its flaws, but it is the best system of governance invented so far<br>8. The rights of minority groups should be protected	Будь ласка, вкажіть, наскільки Ви згодні чи не згодні з цими твердженнями, використовуючи шкалу від 1 – «Абсолютно не згодні» до 7 – «Цілком згодні»:<br>1. Важливо, щоб суди ставилися до всіх однаково<br>2. Громадяни повинні мати право вибирати своїх політичних представників на вільних і чесних виборах<br>3. Право для опозиційних партій вільно критикувати уряд НЕ є важливим<br>4. Судді мають бути незалежними від обраних політиків<br>5. Важливо, щоб ЗМІ могли вільно критикувати уряд<br>6. Право для судів зупинити уряд, якщо він перевищує свої повноваження, НЕ є важливим<br>7. Демократія має свої недоліки, але це найкраща форма державного правління, яку насьогодні винайшло людство<br>8. Слід захищати права груп меншин	Пожалуйста, укажите, насколько Вы согласны или не согласны с этими утверждениями, используя шкалу от 1 – « Совершенно не согласны» до 7 – «Совершенно согласны»:<br><br>1. Важно, чтобы суды относились ко всем одинаково<br>2. Граждане должны иметь право выбирать своих политических представителей на свободных и честных выборах<br>3. Право для оппозиционных партий свободно критиковать правительство не важно<br>4. Судьи должны быть независимы от избранных политиков<br>5. Важно, чтобы СМИ могли свободно критиковать правительство<br>6. Право для судов остановить правительство, если оно превышает свои полномочия, НЕ важно<br>7. Демократия имеет свои недостатки, но это лучшая форма государственного правления, которую сегодня изобрело человечество<br>8. Следует защищать права групп меньшинств
19	Party vote	Which party did you vote for in the last parliamentary elections?<br>1. Opposition Platform — For Life<br>2. Fatherland<br>3. Servant of the People<br>4. European Solidarity<br>5. Voice<br>6. Radical Party of Oleh Lyashko	Q46. За яку партію ви голосували на парламентських виборах у липні 2019?<br><br>1. Опозиційна платформа — за життя<br>2. Батьківщина<br>3. Слуга народу<br>4. Європейська Солідарність	За какую партию вы голосовали на последних парламентских выборах 2019?<br><br>1. Оппозиционная платформа – За жизнь<br>2. Батькивщина<br>3. Слуга народа<br>4. Европейская Солидарность

		7. Other: which one<br>8. Did not vote<br>998 = Prefer not to say	5. Голос<br>6. Радикальна партія Олега Ляшка<br>7. Інша: яка<br>8. Не голосував<br>988 = волію не відповідати на це питання	5. Голос<br>6. Радикальная партия Олега Ляшко<br>7. Другая: какая<br>8. Не голосовал<br>988 = предпочитаю не отвечать на этот вопрос
20	Militaristic Counterattack-scale<br><br>Please present the statements in random order	Please indicate the degree to which you agree with the following statements:<br><br>1. To put an end to Russian war acts, I think it is OK to use enhanced interrogation techniques<br>2. To put an end to Russian war acts, I think it is OK to use torture<br>3. If this would stop the Russian war acts, I think it would be OK to even target civilians on the Russian territory.<br>4. I support continued military efforts to root out Russian soldiers<br>5. We are being way too soft on Russia<br>6. We shouldn't be afraid to hunt down any Russian who threatens our country anywhere<br>7. We should strike back with brutal force against the Russian army<br>8. We should spend more time on diplomatic efforts as opposed to engaging in military activity<br><br>- Strongly Disagree (1)<br>- Disagree (2)<br>- Somewhat Disagree (3)<br>- Neither Agree nor Disagree (4)<br>- Somewhat Agree (5)<br>- Agree (6)<br>- Strongly Agree (7)<br>998 = Prefer not to say		
21	Dehumanization	People can vary in how human-like they seem. Some people seem highly evolved whereas others seem no different than lower animals. Using the image below, indicate with		

	Please present the groups in random order<br><br>The 'evolution image' to be used above the slider will be attached	the slider how evolved you consider the average member of each group to be from 0 = 'Not very well' to 100 = 'Very well' (include 998 = Prefer not to say)<br><br>[Insert image]<br><br>Unmarked slider for evaluation of:<br>1. Russians<br>2. Belarusians<br>3. Ukrainians<br>4. Poles<br>5. French		
22	Infrahumanization<br><br>Please randomize the order of the target group presented for evaluation (Russians and Ukrainians).<br><br>Please randomize the order of emotions for each group.	Please indicate on a slider how well each of the emotions below characterizes the following groups, from 0 = 'Not at all well' to 100 = 'Very well'<br><br>Russians:<br>1. Compassion<br>2. Bitterness<br>3. Pleasure<br>4. Pain<br><br>Ukrainians:<br>1. Compassion<br>2. Bitterness<br>3. Pleasure<br>4. Pain		
23	Identity 1<br><br>Please present the questions in random order	Below is a set of questions related to your personal identification.<br><br>Please answer each of the questions on the scale from 1- Not at all to 7- Very strongly<br><br>1. How strongly do you identify with Ukrainians?<br>2. How strongly do you identify with Russians?		

		3. How strongly do you identify with Europeans?<br><br>Scale:<br><br>1.Not at all<br>2.<br>3.<br>4.<br>5.<br>6.<br>7. Very strongly<br>998 = Prefer not to say		
24	Identity 2<br><br>Please present the questions in random order	Please answer each of the questions on the scale from 1- Not close at all 7- Very close<br><br>1. How close do you feel to Ukrainians?<br>2. How close do you feel to Russians?<br>3. How close do you feel to Europeans?<br><br>Scale:<br><br>1.Not close at all<br>2.<br>3.<br>4.<br>5.<br>6.<br>7. Very close<br>998 = Prefer not to say		
		'This is an attention check. Please pick 'Green' from the list of colors below:<br><br>1) Red<br>2) Blue<br>3) Green<br>4) Orange<br>5) Brown		

25	Comments<br><br>[open question]	This is the last question.<br>Do you have any comments that you would like to share with us?		
26	Debriefing	Thank you very much for your participation.<br><br>The purpose of this research project is to examine how people respond to military attacks and what are their preferences for leaders in conflict situations. Specifically, we want to know if these preferences differ in situations of conflict or peace. We also want to know if people see differences between their ideal leader and their actual leader and what are the consequences of this discrepancy. In addition, we want to investigate if preferences about democracy change during conflict.<br><br>Thank you for your participation in our study. If you have any questions about the survey please contact Honorata Mazepus at h.mazepus@fgga.leidenuniv.nl<br><br>Honorata Mazepus will also disseminate the findings of this study using her Twitter account (@HMazepus).<br><br>In case there are any remaining questions, please feel free to contact me.<br><br>Again thank you very much for your time, participation and effort.<br><br>Dr. Honorata Mazepus<br>Leiden University, the Netherlands		
